# Transcriptome Analysis and VIGS Identification of Key Genes Regulating Citric Acid Metabolism in Citrus

**DOI:** 10.3390/cimb45060295

**Published:** 2023-05-28

**Authors:** Tianxin Chen, Juan Niu, Zhimin Sun, Jing Chen, Yue Wang, Jianhua Chen, Mingbao Luan

**Affiliations:** Institute of Bast Fiber Crops, Chinese Academy of Agricultural Sciences, Changsha 410205, China

**Keywords:** citrus, citric acid, VIGS, WGCNA

## Abstract

Citrus (*Citrus reticulata*) is one of the world’s most widely planted and highest-yielding fruit trees. Citrus fruits are rich in a variety of nutrients. The content of citric acid plays a decisive role in the flavor quality of the fruit. There is a high organic acid content in early-maturing and extra-precocious citrus varieties. Reducing the amount of organic acid after fruit ripening is significant to the citrus industry. In this study, we selected a low-acid variety, “DF4”, and a high-acid variety, “WZ”, as research materials. Through WGCNA analysis, two differentially expressed genes, citrate synthase (CS) and ATP citrate-pro-S-lyase (ACL), were screened out, which related to the changing citric acid. The two differentially expressed genes were preliminarily verified by constructing a virus-induced gene-silencing (VIGS) vector. The VIGS results showed that the citric acid content was negatively correlated with CS expression and positively correlated with ACL expression, while CS and ACL oppositely control citric acid and inversely regulate each other. These results provide a theoretical basis for promoting the breeding of early-maturing and low-acid citrus varieties.

## 1. Introduction

Citrus (*Citrus reticulata*) is one of the world’s most widely planted and highest-yielding fruit trees. Citrus fruits are rich in various nutrients, such as carbohydrates, vitamins, antioxidants, and mineral elements [1]. The flavor of citrus fruits is the main selection criterion for the consumer market. Organic acids, soluble sugars, and aromatic substances are the key factors forming the fruit flavor quality. The sugar and acid content of the fruit, especially the level and proportion of sugar and acid components, ultimately determine the flavor of the fruit [2]. With the development of the citrus industry, some problems have gradually appeared in the structure of the citrus industry. Currently, the main citrus varieties in China are mainly mid-maturing, saturating the market from November to December. A few extra-precocious and early-maturing varieties mature before October, and the late-maturing varieties ripen from February to September the following year [3]. There is a problem with the high organic acid content in early-maturing and extra-precocious citrus varieties, which lengthens the fruit storage cycle and lags behind the marketing time of citrus. Therefore, reducing the content of organic acids after fruit ripening is significant when adjusting the citrus market structure.

There are many kinds of organic acids in fruits, which vary from fruit to fruit. However, most fruits contain one or two main organic acids, and a few others exist in small amounts. According to the types of organic acids that are mainly formed and accumulated during fruit growth and development, fruits can be divided into three acid types: malic acid, tartaric, and citric acid [4]. Citrus fruit is a typical citric acid fruit. Yamaki et al. determined 48 citrus varieties regarding the type and content of organic acid. The results showed that citric acid had the highest content in most citrus varieties, at a rate of about 75.40%, comprising 96.90% of the total organic acid content, followed by malic acid [5]. The changes in the accumulation of organic acids in different varieties or the growth and development stages of the same type are inconsistent, and the opposite situation can occur [6]. As a typical citric acid fruit, the change in organic acid content in fruit mainly depends on the level of synthesis and accumulation of citric acid. For most citrus fruits, rapid synthesis and the accumulation of citric acid occur at the early stage of fruit development, reaching a relatively high level. However, with the gradual ripening of the fruit, the citric acid content shows a gradual downward trend [7]. Citric acid accumulation in citrus fruits is a complex process, and its content is affected by citric acid synthesis, degradation, storage, transport, and other aspects. Previous studies and reports have agreed on the metabolic pathway, accumulation mode, and theory of citric acid in citrus [8]. However, the key metabolic pathways affecting citric acid content in fruits need further research and verification [9]. Many studies on the decomposition and consumption of citric acid in citrus fruits are available, and most reports support the idea that citric acid decomposition and utilization directly affect citric acid content. In addition to the classical tricarboxylic acid cycle (TCA) pathway, the Gamma-aminobutyric acid (GABA) pathway and citric acid cleavage pathway have also been reported to be involved in regulating citric acid content in citrus fruits, but the main regulatory pathways or regulatory genes in these pathways need to be further clarified [10]. Gene differences between varieties can be assessed by transcriptome sequencing. 

Virus-induced gene silencing (VIGS) is a plant RNA-silencing technique that uses viral vectors carrying the target gene fragment to induce endogenous gene silencing. The function of the target genes can be assessed according to the phenotypic variation [11]. Compared with the traditional gene function analysis methods, VIGS can silence and analyze the target genes in infected plants, avoid plant genetic transformation, play a role in different genetic backgrounds, and analyze the gene function more thoroughly [12]. The technical operation is simple, and the research cycle is short. Generally, one test cycle can be completed in 1–3 weeks. This has been used to study the functional genes of disease resistance, growth and development, and metabolic regulation in tobacco, tomato, and other plants [13,14,15]. It is often used in citrus leaves but rarely used in fruits.

In this study, RNA sequencing data were used to reveal the key differential genes regulating the citric acid metabolism pathway between Dafen 4 (low-acid variety) and Weizhang (high-acid variety) at 66–136 days after flowering in six developing stages. In addition, we preliminarily verified the key differential genes using the VIGS technique. This study aims to provide a theoretical basis for understanding the molecular mechanism of citric acid metabolism.

## 2. Materials and Methods

### 2.1. Plant Material

We selected two kinds of citrus materials for this experiment: low-acid-content citrus (DF4) and high-acid-content citrus (WZ). The details of the plant materials were explained in our previous report [16,17]. The plants were cultivated in the experimental field of the Institute of Bast Fiber Crops, Chinese Academy of Agricultural Sciences (Yuanjiang, China). Organic acid metabolic and variations in sugar content were evaluated, and the sample collection time ranged from July to September 2021. The fruit was harvested on the 66 DAF (the day after flowering) and then collected every 14 days until 136 DAF. During the collection, four fruits located in the northwest and southeast were collected as a sample, and each sample had three biological repeats. The collected part was put into liquid nitrogen and stored in an ultra-low freezer (−80 °C) for transcriptome sequencing, and the other part was used for quality analysis.

### 2.2. Measurement of Total of Soluble Sugar (TSS) Titratable Acid (TA) in Citrus Flesh

After being mixed well, a refractometer was used to measure TSS in the collected samples. TA was measured using titrimetric analysis, using 0.1 mol/L NaOH for neutralization [18].

### 2.3. Quantification of Organic Acids and Soluble Sugars in Citrus Fruit Flesh

The citrus pulp was ground with liquid nitrogen and mixed with 80% ethanol for ultrasonic extraction. The citric acid and malic acid contents were determined using high-performance liquid chromatography (HPLC, Agilent 1260, Santa Clara, CA, USA) with 2% methanol and 98% K_2_HPO_4_ used as the mobile phase and 0.6 mL/min as the flow rate. The glucose, fructose, and sucrose content were determined with the anthrone sulfuric acid method and spectrophotometer (SV, MAPADA UV-3000, Beijing, China).

### 2.4. RNA Extraction and Transcriptome Sequencing Data Comparison

Total RNA was extracted from the six stages of development (66, 80, 94, 108, 122, and 136) DAF using the RNA prep Pure Plant kit (Tiangen, Beijing, China). Then, using NanoDrop Agilent 2100 bioanalyzer (Thermo Fisher Scientific, Waltham, MA, USA), the purity of the RNA sequencing of transcriptome library through Huada Gene company (Illumina, Beijing, China) was measured. Then, the remaining clean reads were aligned by reference Citrus clementina genome (Citrus clementina v1.0) using HISAT2 (v2.1.0) [19], from which unigenes were obtained.

### 2.5. Analysis and Enrichment of Differential Expression Genes

The differentially expressed genes (DEGs) were identified with the DESeq2 R package [20]; statistical significance was defined using Benjamini–Hochberg-adjusted *p*-values < 0.05. After the pairwise comparison of different varieties in the same period and different periods of the same variety, the differentially expressed genes were obtained. GO, and KEGG enrichment was performed with the Clusterprofiler R package [21].

### 2.6. WGCNA for Identifying Hub Genes and Weight Modules 

Weighted gene co-expression network analysis (WGCNA) was performed in R to rank genes into coexpressed modules. FPKM values were normalized, and a nearness matrix was made. The quality data and FPKM were imported into R, and correlation-based associations between quality data and factor modules were calculated [22,23]. After the structure topological overlap matrix (TOM), transcripts with the same expression pattern were divided into a module, and the characteristic genes of these modules were calculated together.

### 2.7. Validation of Intramodular Candidates through RT-qPCR Analysis

Quantitative reverse transcription-PCR (RT-qPCR) was employed to validate the RNAseq data. Total RNA extracted from 36 samples was reverse-transcribed to cDNA using the PrimeScript RT Master Mix for qPCR (Takara, Dalian, China). The sequences of specific primers for the internal control and differentially expressed genes are shown in Table S1. Relative expression levels were calculated using the method used in the previous report [24].

### 2.8. Construction of the VIGS Vector

Using the candidate gene coding frame (300–500 bp) and the polyclonal restriction site of the VIGS vector with Tobacco rattle virus (TRV), primers were designed, and the recombinant DNA was constructed with cDNA as a template. The recombinant plasmids DNA and TRV2 vector were digested with a restriction endonuclease. The target fragments were recovered, ligated with T4DNA ligase, and transformed into *E. coli* competent cells. The recombinant plasmids were screened with kanamycin (Kan^+^). PCR and sequencing identified the transformation results. The correct plasmids were transformed into Agrobacterium tumefaciens competent cells. The recombinant plasmids were screened using a kanamycin (Kan^+^) resistance/rifampicin (rif) plate [25]. The transformation results were identified by PCR and sequencing.

### 2.9. Infection in Citrus Fruit with VIGS Vector

The vector transferred into *Agrobacterium* was used for the expanded culture. When the OD value of Agrobacterium tumefaciens was 2.5–2.7, 10 mL of the bacterial solution was obtained; this was centrifuged at 4500 rpm speed for 10 min, and the supernatant was discarded. Then, the sediment was mixed by resuspending (10% MES,10% MgCl_2_,80% ddH_2_O). Then, 10 μL AS (10 mg/mL) was added, TRV1 and candidate genes were mixed at 1:1, and TRV1 and TRV2 1:1 were mixed and placed in an ice box. A 5 mL disposable syringe was used to inject the top of the fruit with *Agrobacterium* tumefaciens (the fruit can be injected in multiple locations). Three biological repeats were selected for each variety, and two fruits were chosen for injection at two locations for each repetition [26]. After infection for 14 days, fruits were collected for further processing.

### 2.10. Validation of Quality Traits and VIGS Infection 

The methods described above were used to determine the TA, citric acid, and malic acid after fruit picking. Similarly, the above-described method was used to extract RNA and reverse-transcribe it into cDNA. All the cDNAs were verified by 1% agarose gel electrophoresis with the VIGS primers. The correct cDNA was validated using RT-qPCR.

## 3. Results

### 3.1. Variations among TSS, TA, and TSS/TA during Citrus Fruit Flesh Development

**Figure 1:**
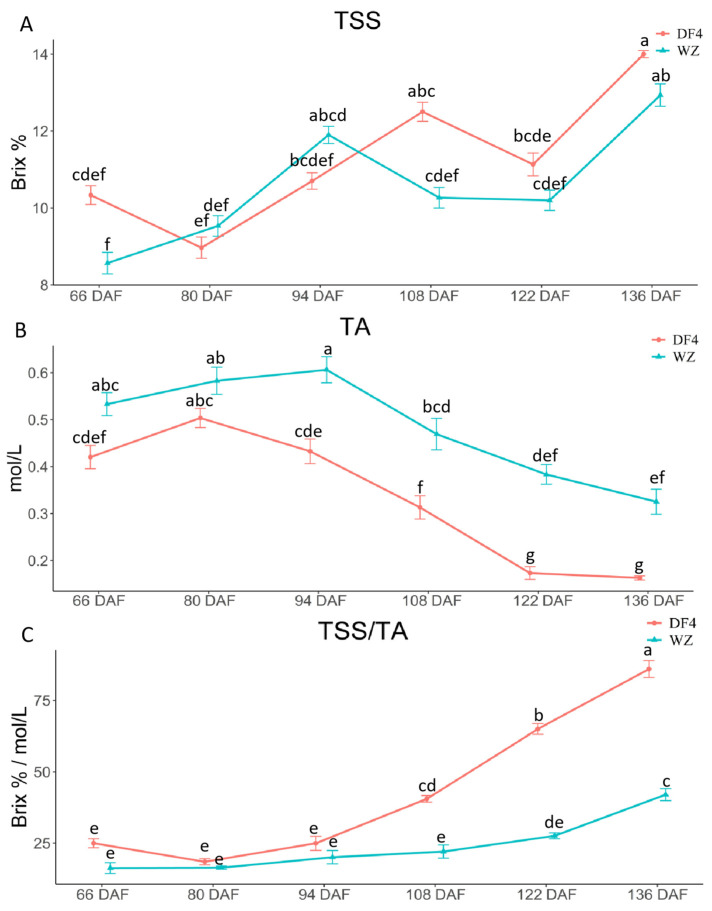
TSS (Brix %), TA (mol/L), and TSS/ TA in citrus fruit at 66–136 DAF. (**A**) TSS in citrus fruit; (**B**) TA in citrus fruit; (**C**) TSS/ TA in citrus fruit. “a, b, c, d, e, f” indicates the statistical significance of the data; “a” was the most significant. Other letters decrease in alphabetical order.

During fruit development, TSS, TA, and TSS/TA can reflect the flavor quality. Figure 1A showed that, with changes in the developmental stage, the TSS of DF4 and WZ showed an overall upward trend, and both reached the maximum value at 136 DAF, but there was no significant difference between the varieties. In Figure 1B, changes in the developmental stage showed that the TA of DF4 and WZ initially increased and then decreased. The TA inflection point of DF4 was 14 days earlier than that of WZ, and the TA of DF4 was significantly lower than that of WZ. Figure 1C showed that the TSS/TA of the two varieties indicated a gradual upward trend with the developmental stage. TSS/TA in DF4 increased significantly more after 108 DAF than in WZ. The results showed that the main factor affecting quality traits in the two varieties was TA, and the TSS had no significant effect on quality traits.

### 3.2. Variations of Minalic Acid and Citric Acid during Citrus Fruit Flesh Development

**Figure 2:**
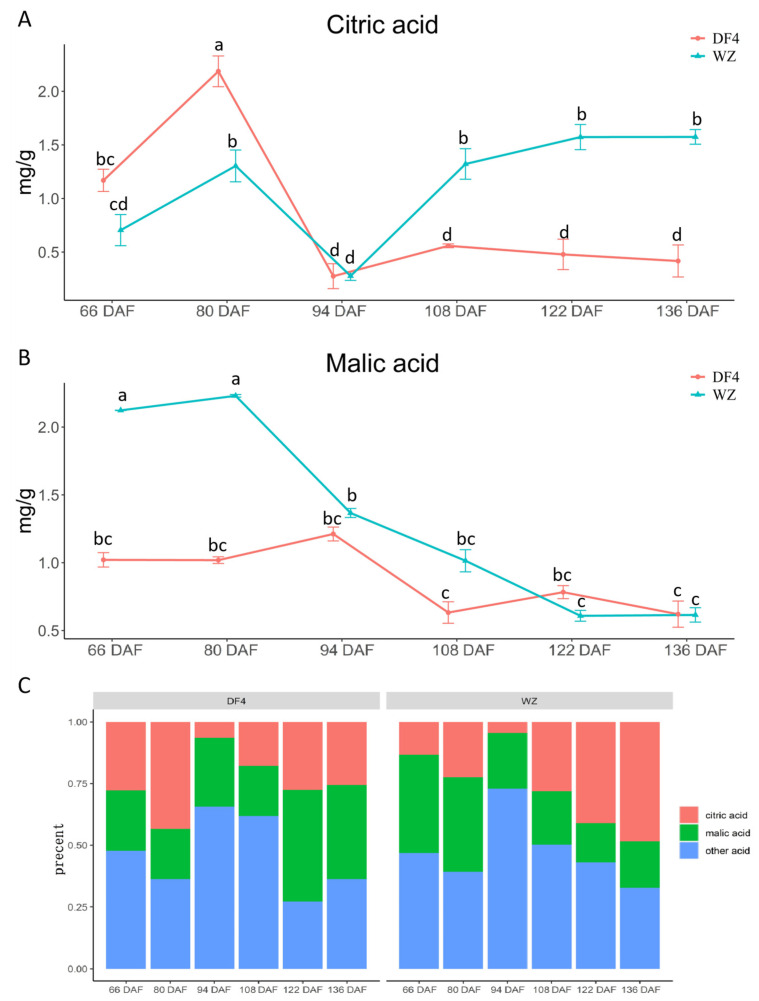
Malic acid and citric acid (mg/g) in citrus fruit at 66–136 DAF. (**A**) Malic acid in citrus fruit; (**B**) Citric acid in citrus fruit; (**C**) Malic acid and citric acid percentages of TA in citrus fruit. “a, b, c, d” indicates the statistical significance of the data; “a” was the most significant. Other letters decrease in alphabetical order.

Malic acid and citric acid mainly affected the acid content of the fruit. A significant difference was found in the citric acid content between DF4 and WZ. Figure 2A shows that the citric acid content of DF4 initially increased and then decreased as the developmental stages changed. The citric acid content of WZ was lower during early growth and higher in the later stage. As shown in Figure 2B, the malic acid content of the citrus fruits showed an overall downward trend as the plants developed. The malic acid content in WZ was higher in the early growth stage. Still, it decreased rapidly at 80 DAF, and there was a significant difference between periods. In comparison, the malic acid content in DF4 was lower in the early stage and slowly decreased at 94 DAF, and there was no significant difference between the periods. As shown in Figure 2C, citric acid, and malic acid are dominant. In the late developmental stage (108 DAF–136 DAF), the citric acid percentage of low-acid-variety DF4 was lower than that of high-acid-variety WZ, but the malic acid proportion of low-acid-variety DF4 was higher than that of high-acid-variety WZ.

### 3.3. Variations in Fructose, Sucrose, and Glucose during Citrus Fruit Flesh Development

**Figure 3:**
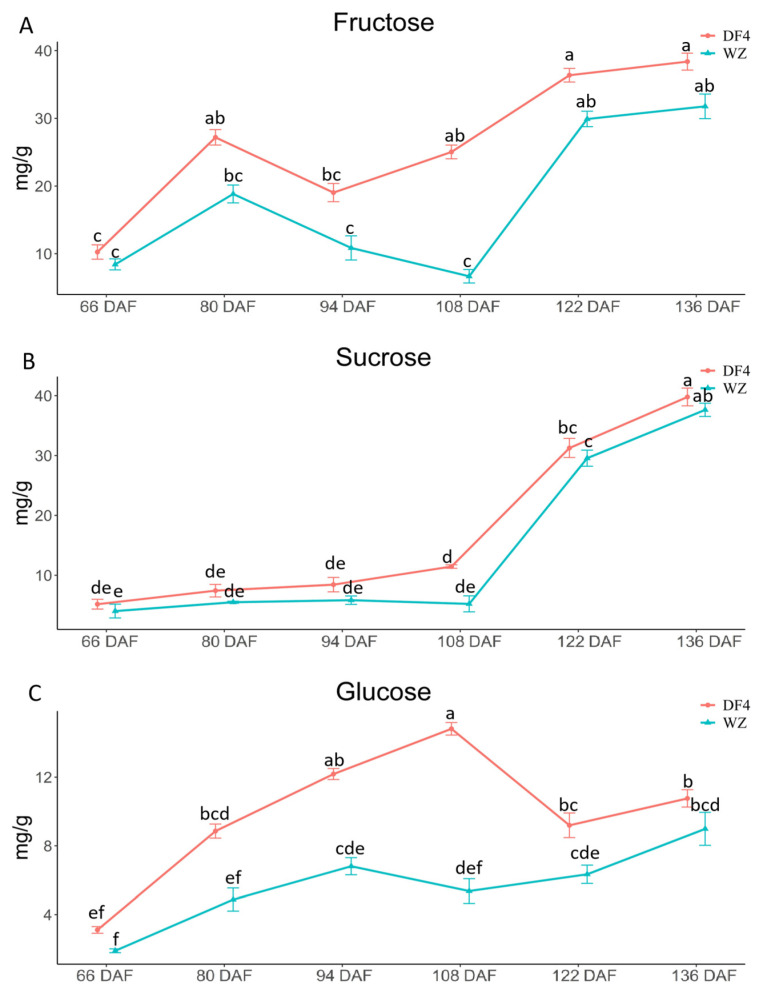
Fructose, sucrose, and glucose content (mg/g) in citrus fruit at 66–136 DAF. (**A**) Fructose in citrus fruit; (**B**) Sucrose in citrus fruit; (**C**) Glucose in citrus fruit. “a, b, c, d, e, f” indicates the statistical significance of the data; “a” was the most significant. Other letters decrease in alphabetical order.

The fructose content of the citrus fruit showed an upward trend as the plants developed (Figure 3A). The fructose content of the two varieties was lower in the early stage. The DF4 fructose content continued to increase at 94 DAF, while that of WZ continued to rise at 108 DAF. Figure 3B shows that, with changes in growth, the sucrose content of the citrus fruits showed an upward trend. The sucrose content of the two varieties remained low in the early stage and rapidly increased at 108 DAF. The sucrose content of DF4 and WZ reached the maximum at 136 DAF. During the development period, the citrus fruit’s sucrose content initially increased, then decreased and increased again (Figure 3C). The glucose content of the two varieties was lower in the early stage. DF4 glucose content decreased at 108 DAF and further increased at 122 DAF. The WZ glucose content decreased at 94 DAF and rose again at 108 DAF. There were significant differences in fructose at 108 DAF between the two varieties. With the continuous immaturity of fruit, the sugar content of the two varieties gradually increased, but there was no significant difference, which indicated that fructose, sucrose, and glucose were not the main factors leading to the difference between low-acid varieties and high-acid varieties.

### 3.4. Identification and Analysis of DEGs at Different Fruit Developmental Stages

**Figure 4:**
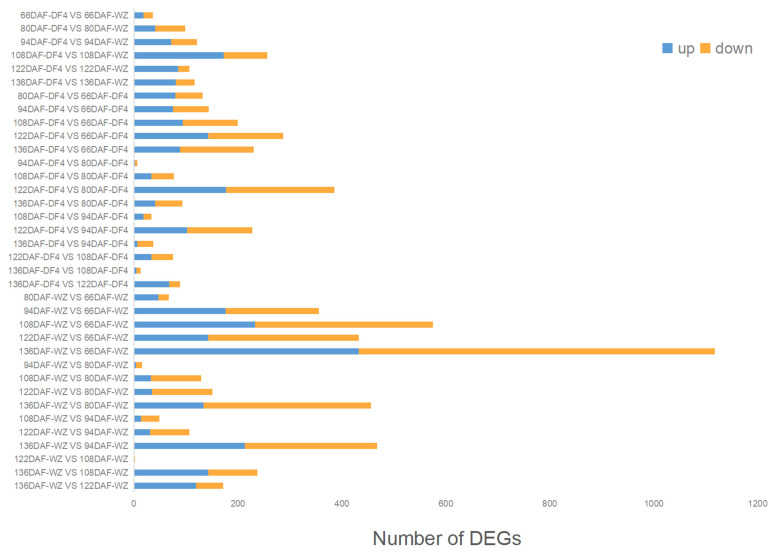
DEGs between six fruit developmental stages of the two samples. Pairwise comparisons of gene expression levels at different stages within each sample and gene expression levels at the same stage between the two samples.

After analyzing the citrus fruit quality traits, two varieties, DF4 and WZ, were selected, including six developmental stages, three biological repeats, and 36 samples for transcriptome sequencing. After removing the low-quality reads and joint sequences, 796 million clean reads were obtained, with a Q20 base of 95.75–96.67% and a GC content of 45.59–46.81% (Table S2). After using the hisat2 comparison and htseq-count quantification, 16310 gene read counts were obtained. As seen in Figure 4, a total of 7105 DEGs were identified using a pairwise comparison of different stages and the same stage in each sample.

### 3.5. Obtaining Hub Genes during Weighted Correlation Network Analysis

**Figure 5:**
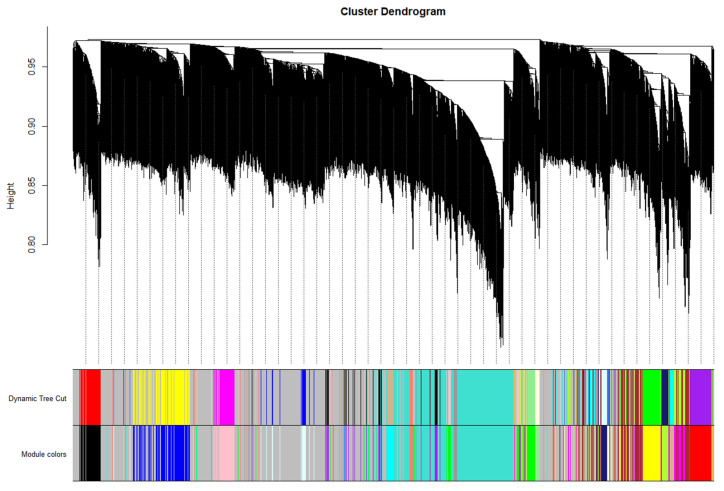
WGCNA quality traits Cluster Map. The result of quality data and cluster analysis, through which quality trait data were grouped into different color modules according to their clustering results as representatives.

**Figure 6:**
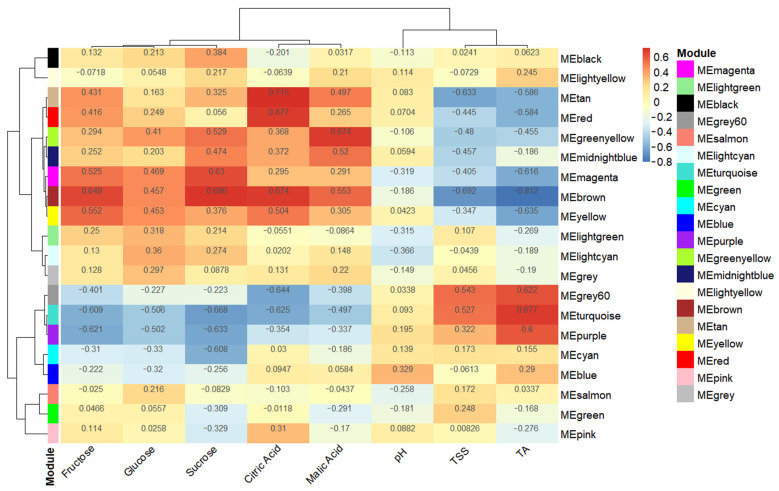
Heat map of correlation between WGCNA quality traits and differentially expressed genes. The module with the highest correlation degree was chosen (the color in the figure is red to indicate a correlation degree > 0.7). Figures on the heat map denote correlation and *p*-value between module and traits. In each module, the upper number represents correlation; the lower number represents *p*-value.

**Figure 7:**
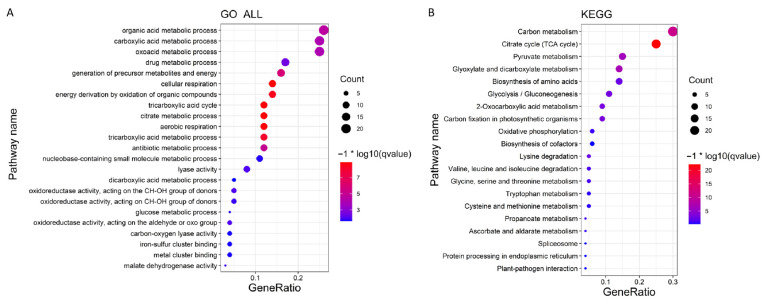
Module MEtan enrichment diagram. (**A**) GO enrichment analysis; (**B**) KEGG enrichment analysis. −1 * log10 means the log transformation of qvalue.

**Figure 8:**
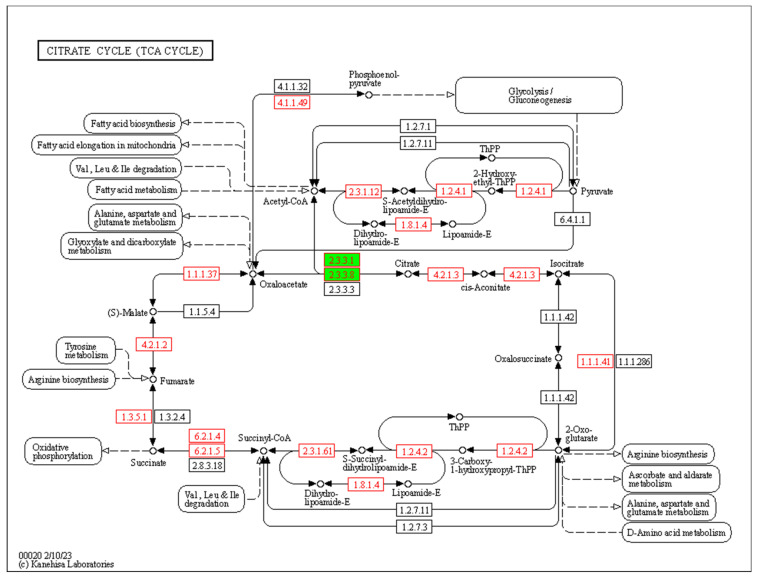
Candidate genes are involved in the TCA pathway metabolic processes.

A correlation analysis was conducted between the 7105 DEGs obtained from the transcriptome data analysis and quality trait data. Figure 5 shows the regulatory genes related to quality traits, classified into several modules; different colors represent different modules. In Figure 6, the module MEtan is the weight module of the citric acid change, with a correlation between module MEtan and citric acid of higher than 0.7. Figure 7 shows that, after gene enrichment analysis, hub genes in the module MEtan were mainly enriched in terms of the organic acid metabolic process and the TCA cycle pathway. The hub genes that were enriched in the pathway were used as candidate genes for the next analysis. The main direction of this study was to mine the genes regulating citric acid metabolism. The tricarboxylic acid cycle pathway is the main pathway of citric acid metabolism; therefore, we screened the weight genes that were enriched in the tricarboxylic acid cycle. The screened genes are shown in Table S3. In Figure 8, the red box represents the candidate genes that were enriched in the tricarboxylic acid cycle. While enriching the candidate genes, we found that the candidate genes Ciclev10031423m.1 and Ciclev10031633m.1 are also enriched at the key enzymes of citrate metabolism (represented by the green module in Figure 8).

### 3.6. Screening Candidate Genes via RT-qPCR

**Figure 9:**
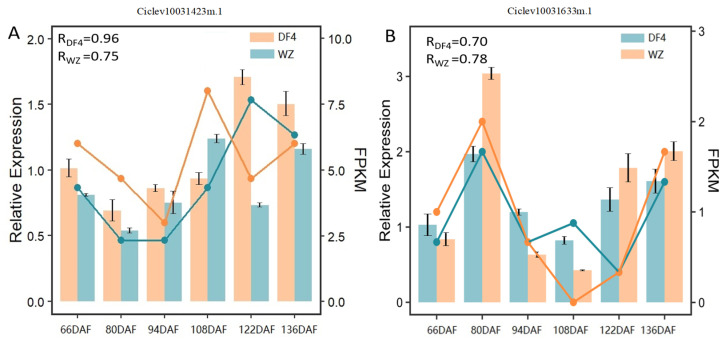
RT-qPCR verification and FPKM results of candidate expression genes. R means Pearson correlation coefficient (PCC) between relative expression and FPKM. (**A**) RT-qPCR verification and FPKM results of CICLE_v10031423mg (CS); (**B**) RT-qPCR verification and FPKM results of CICLE_v10031633mg (ACL). The bar graph shows gene RT-qPCR values, and the line graph shows the gene FPKM.

Bioinformatics analyses were used to enrichen differentially expressed genes in the module MEtan, which directly regulates citric acid content in the tricarboxylic acid cycle. RT-qPCR primers were designed for the differentially expressed genes and verified with RT-qPCR. After combining these with quality trait data, it was found that the RT-qPCR results of two differentially expressed genes, Ciclev10031423m.1 (CS:citrate synthase) and Ciclev10031633m.1 (ACL:ATP citrate (pro-S)-lyase), were consistent with the changing trend of FPKM. The correlation between relative expression value and FPKM was evaluated by calculating the Pearson correlation coefficient (PCC). The PCC values of these two genes were higher than 0.7. The gene expression trends of RNA-seq and RT-qPCR results were highly consistent (see Figure 9). These two genes (CS and ACL) were used as candidate genes for further analysis.

### 3.7. Construction of VIGS Vector and Verification of Impregnation Result

**Figure 10:**
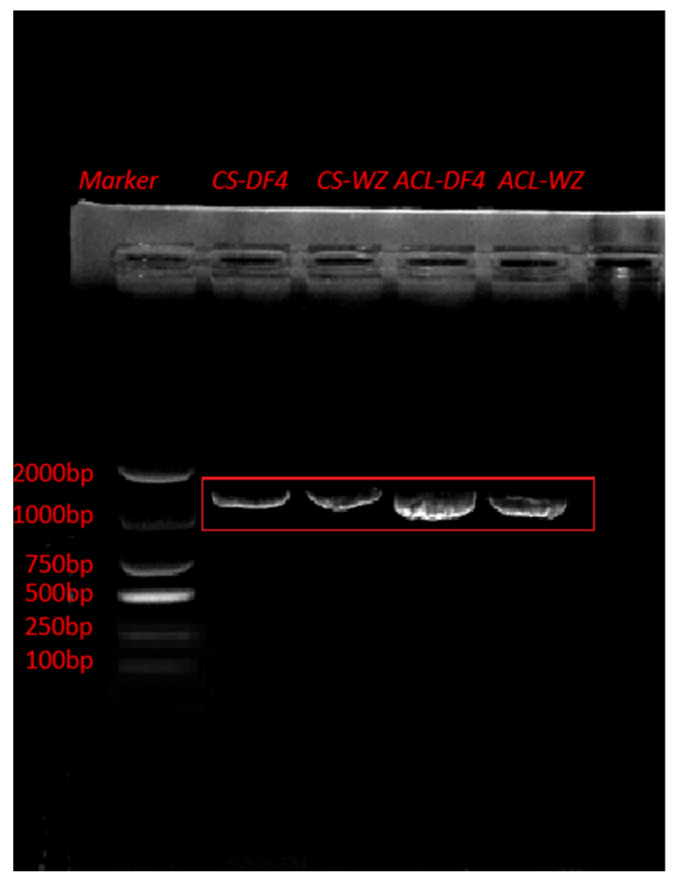
PCR verification with CS and ACL. The red box indicated correct site.

**Figure 11:**
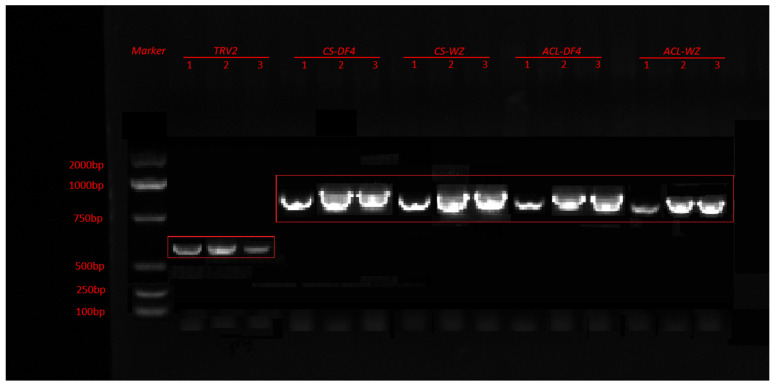
PCR verification with TRV2, VIGS-CS, and VIGS-ACL. The red box indicated correct site.

**Figure 12:**
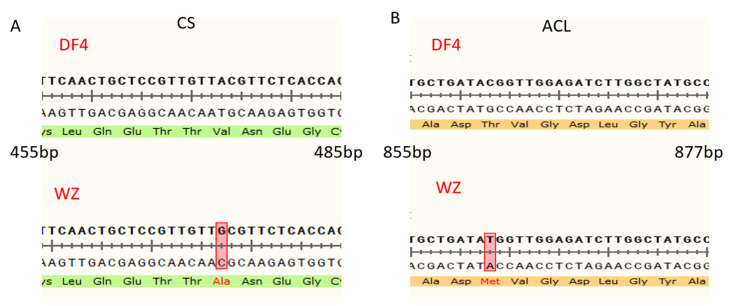
Sequence alignment between DF4 and WZ in CS and ACL. (**A**) Sequence alignment between DF4 and WZ in CS; (**B**) Sequence alignment between DF4 and WZ in ACL. The red box indicated the location of the base mutation.

The PCR products were verified with 1% agarose gel electrophoresis. Figure 10 shows that CS and ACL were successfully amplified in DF4 and WZ with lengths of 1407 bp and 1272 bp, respectively. In this figure, ACL-DF4 is Thr, and ACL-WZ is Met. VIGS vectors were detected in all the infected fruits, indicating that VIGS vectors were successfully transferred into citrus fruits (Figure 11). After comparing the amplified sequences of the two varieties, Figure 12A shows that CS has a single base mutation at 473 bp, which changes the amino acid sequence. In this figure, CS-DF4 is Val, and CS-WZ is Ala. Figure 12B shows that ACL has a single base mutation at 863 bp, which changes the amino acid sequence. 

### 3.8. Effect of VIGS Infection on the Content of RT-qPCR Citric Acid and TA

**Figure 13:**
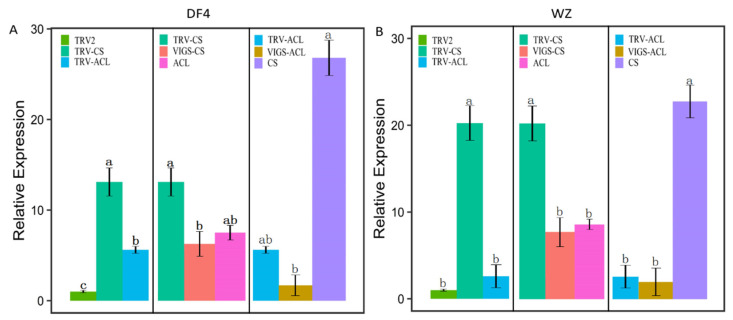
RT-qPCR verification of candidate gene expression with VIGS. (**A**) RT-qPCR verification of candidate gene expression in DF4; (**B**) RT-qPCR verification of candidate gene expression in WZ. TRV2 (Empty vector as a control). TRV-CS/TRV-ACL (the amount of CS/ACL gene expression in fruits without VIGS infection as a control). VIGS-CS/VIGS-ACL (the amount of CS/ACL gene expression after fruit infection). ACL (the expression of ACL gene in fruits silenced with CS). CS (the expression of CS gene in fruits with silenced ACL). “a, b, c” indicates the statistical significance of the data; “a” was the most significant. Other letters decrease in alphabetical order.

**Figure 14:**
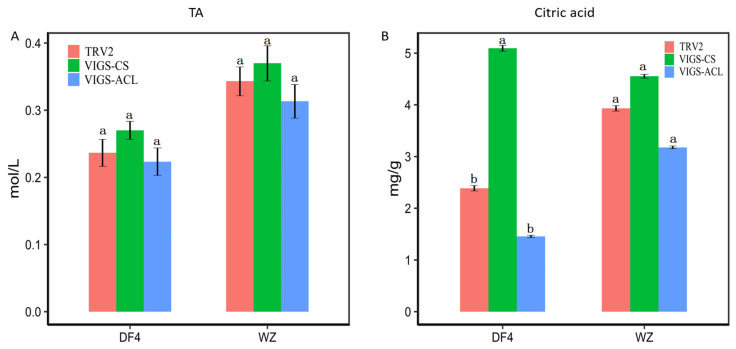
TA (mol/L) and Citric acid (mg/g) in citrus fruit with VIGS. (**A**) TA in citrus fruit; (**B**) Citric acid in citrus fruit. “a, b” indicates the statistical significance of the data; “a” was the most significant. Other letters decrease in alphabetical order.

Both the RT-qPCR of CS and ACL were measured in VIGS-CS and VIGS-ACL fruits. Figure 13A showed that, after VIGS insertion in DF4, the expression of CS and ACL decreased compared with the control group, which indicated that VIGS had an effect. The expression of ACL increased after silencing CS, and after silencing ACL, the expression of CS significantly increased. Figure 13B shows that, after VIGS insertion in WZ, the two varieties showed the same trend. This result suggests that CS and ACL may influence each other. Figure 14A shows that silencing CS increases TA compared with the control TRV2 while silencing ACL decreases TA. Compared with the control TRV2, silencing CS increased citric acid content, while silencing ACL decreased citric acid content (Figure 14B). Both DF4 and WZ show the same trend. In sum, CS reversely regulates citric acid content for TA, while ACL positively regulates TA and citric acid content.

## 4. Discussion

Soluble sugars in citrus mainly exist as fructose, sucrose, and glucose, among which the sucrose content is the highest [27,28]. Studies have shown that the ratio of sucrose, glucose, and fructose in some early- and medium-maturing citrus fruits is mainly 2:1:11 [29]. Related studies showed no significant difference in sugar content between wild and cultivated citrus fruits, indicating no substantial change in the soluble sugar content during citrus domestication [30]. During the development of the citrus fruit, the content of soluble sugar in the juice sacs gradually accumulated with fruit development, and the accumulation rate of sucrose was higher than that of glucose and fructose [31]. Great differences in soluble sugar content among different citrus varieties have been reported [32]. In this study, the soluble sugar content of DF4 and WZ gradually increased during the growth period, consistent with previous studies. At 150 DAF, the proportion of fructose, sucrose, and glucose in DF4 is about 3%, 4%, and 1%. In WZ, the ratio of fructose, sucrose, and glucose is about 4.5%, 5.3%, and 5.3%. Compared with previous studies, the ratio of the glucose content is relatively consistent, but the ratio of fructose to sucrose is different. This showed differences in the content and proportion of soluble sugars in citrus fruits among different varieties and growth stages.

While the content of soluble sugars in citrus fruit is high, the content of organic acid in the fruit has become the key factor affecting the quality of citrus fruit. In the early-ripening variety Wenzhou mandarin, citric and malic acid increased at the same rate in the initial stage of fruit growth and development, and their contents were almost the same. However, with fruit growth, the content of citric acid rapidly increased, reaching its peak in September and then gradually decreasing; its accumulation was much higher than that of malic acid [33]. The research found that the organic acid content of Clemens citrus reached its peak 137 days after flowering and then gradually decreased [34]. However, some studies have found that the change in the organic acid content of some citrus fruits differs from that of most citrus fruits with the growth and development of the fruits, but this showed the opposite trend. The organic acid content gradually increased or maintained a low level during the whole process of growth and development. For example, the citric acid content in high-acid citrus and high-acid lemon fruits gradually increased during growth and development. The citric acid content of high-acid lemon peaked 150 days after flowering, and the citric acid content of high-acid lemon reached a peak 100 days after flowering. The citric acid content of both remained at a high level during growth and development. The citric acid in acid-free citrus and acid-free lemon fruits remained stable at a low level during the growth and development process [35]. In fruit growth, development, and ripening, the citric acid content of red willow, acid-free pomelo, and other varieties remained at a low level [36]. In this study, DF4 and WZ reached their inflection point at 80 DAF and their minimum at 94 DAF. Overall, a trend of higher acid content in the early stage and lower acid content in the later stage was maintained, which was consistent with previous studies, but the acid reduction period was relatively early compared with other varieties. This showed that the acid reduction inflection point of citrus fruit was substantially different among varieties.

The citric acid in citrus fruit is the intermediate product of the TCA cycle. Studies have shown that citrus fruits can independently carry out photosynthesis before changing color [37], which can provide substrates for the tricarboxylic acid cycle. On the other hand, studies have shown that citrus leaves affect citric acid accumulation in fruits [38], but there is little direct evidence for citric acid’s transport from roots or leaves into fruits. Citrate synthase (CS) is one of the most important enzymes in the tricarboxylic acid cycle, which catalyzes the combination of oxaloacetic acid (OAA) and acetyl-coenzyme A (acetyl-CoA) to form citric acid [39]. Early studies showed that spraying arsenate inhibited CS activity and decreased citric acid content in citrus fruits, indicating that CS plays an important role in citric acid accumulation in fruits [40]. However, many reports showed no significant correlation between CS and citric acid accumulation. Shi et al. [41] studied the relationship between CS gene expression and pomelo fruit acid content and concluded that it had no significant correlation with the high- and low-citric-acid phenotypes of pomelo fruit. The AN analysis of the gene expression and enzyme activity of three fruits, sweet lemon, sour lemon, and “Shamoti sweet orange”, with different acidity levels also showed that the difference in citric acid content was not directly related to the change in CS [42]. Some reports showed that spray phosphate reduced CS activity and, thus, citrate content. The study of Chen et al. [43] focusing on lemon, Jin orange, Bingtang orange, and Fengjie navel orange also showed no significant relationship between a change in CS activity and the difference in citric acid content in different types of citrus fruits. Therefore, although CS is an important enzyme directly involved in citric acid synthesis, its role in citric acid accumulation in fruit remains unclear. The Ciclev10031423m.1 (CS) gene, regulating CS, was identified in this study. The expression of Ciclev10031423m.1 (CS) in DF4 and WZ initially decreased and then increased with the growth period, contrary to the citric acid content trend. To some extent, CS may reverse regulate the activity of the CS enzyme, thus affecting the change in citric acid content in citrus fruit. ATP-citrate lyase (ACL) exists in the cytoplasm, catalyzes the decomposition of citrate into acetyl-CoA and oxaloacetic acid, and also affects the accumulation of citric acid [44]. Katz et al. [45] reported that ACL was involved in citric acid cleavage during fruit-ripening in Washington navel oranges, while research showed that the downregulation of ACL gene expression could lead to citric acid accumulation, and its expression had slightly different effects on the citric acid content in different citrus fruits [46]. The Ciclev10031633m.1 (ACL) gene regulating ACL was also identified in this study. The expression of Ciclev10031633m.1 (ACL) in DF4 and WZ first increased and then decreased with the growth period, which was the same as the changing trend of citric acid content. This indicates that ACL may positively regulate the activity of ACL enzyme, thus affecting the change in citric acid content in citrus fruit.

Currently, the establishment of the VIGS system is reported more in citrus leaves than in citrus fruits. In this study, VIGS vectors for two candidate genes were constructed and successfully transferred into fruits. After silencing, the expression of the two candidate genes was downregulated, which confirmed the reliability of VIGS. Compared with the control group, the changes in the TA and citric acid content of TRV2, CS, and ACL silencing were opposite. The results further verified the regulatory effect of candidate genes on citric acid. It was found that the expression of ACL increased in CS silencing, and the expression of CS increased in ACL silencing and was consistent in DF4 and WZ. These results indicated that the expression of CS and ACL might influence and regulate each other in reverse.

At present, the main citrus varieties are still mid-maturing varieties from November to December, and relatively few early-maturing citrus varieties mature before October. In addition, the phenomenon of high citric acid content is common in early-maturing varieties, so the breeding of low-acid and early-maturing citrus varieties can not only meet the needs of consumers but also expand and regulate the citrus market and promote the healthy development of the citrus industry [47,48,49]. The results of this study provide theoretical support for the breeding of low-acid and early-maturing citrus varieties and the development of molecular markers.

## 5. Conclusions

In this study, two differentially expressed genes related to the change in citric acid content using transcriptome and quality trait data were screened. The two differentially expressed genes of CS and ACL were preliminarily verified by constructing a VIGS vector. The results showed that the two differentially expressed genes could regulate the change in citric acid content. Through VIGS, we found that CS and ACL oppositely regulated citric acid and controlled each other inversely. In this study, candidate genes for regulating citric acid content during citrus fruit development were identified and preliminarily verified via VIGS. These results provide a theoretical basis for promoting the breeding of early-maturing and low-acid citrus varieties.

## Data Availability

All data needed to evaluate the conclusions in this paper are present in the paper and/or the Supplementary Materials.

## Supplementary Materials

The following supporting information can be downloaded at: https://www.mdpi.com/article/10.3390/cimb45060295/s1, Table S1: Primers for candidate genes of RT-qPCR; Table S2: Quality control results of transcriptome sequencing machine data; Table S3: Candidate genes in the TCA cycle.
